# Management of brain metastasis. Surgical resection versus stereotactic radiotherapy: a meta-analysis

**DOI:** 10.1093/noajnl/vdac033

**Published:** 2022-03-09

**Authors:** David T Krist, Anant Naik, Charee M Thompson, Susanna S Kwok, Mika Janbahan, William C Olivero, Wael Hassaneen

**Affiliations:** 1 Department of Neurosurgery, Carle Foundation Hospital, Urbana, Illinois, USA; 2 Carle Illinois College of Medicine, Champaign, Illinois, USA; 3 Department of Communication, University of Illinois at Urbana-Champaign, Urbana, Illinois, USA

**Keywords:** brain metastasis, local tumor control, meta-analysis, surgical resection, stereotactic radiotherapy

## Abstract

**Background:**

Treatment of metastatic brain tumors often involves radiotherapy with or without surgical resection as the first step. However, the indications for when to use surgery are not clearly defined for certain tumor sizes and multiplicity. This study seeks to determine whether resection of brain metastases versus exclusive radiotherapy provided improved survival and local control in cases where metastases are limited in number and diameter.

**Methods:**

According to PRISMA guidelines, this meta-analysis compares outcomes from treatment of a median number of brain metastases ≤ 4 with a median diameter ≤ 4 cm with exclusive radiotherapy versus surgery followed by radiotherapy. Four randomized control trials and 11 observational studies (1693 patients) met inclusion criteria. For analysis, studies were grouped based on whether radiation involved stereotactic radiosurgery (SRS) or whole-brain radiotherapy (WBRT).

**Results:**

In both analyses, there was no difference in survival between surgery ± SRS versus SRS alone two years after treatment (OR 1.89 (95% CI: 0.47–7.55, *P* = .23) or surgery + WBRT versus radiotherapy alone (either WBRT and/or SRS) (OR 1.18 (95% CI: 0.76–1.84, *P* = .46). However, surgical patients demonstrated greater risk for local tumor recurrence compared to SRS alone (OR 2.20 (95% CI: 1.49–3.25, *P* < .0001)) and compared to WBRT/SRS (OR 2.93; 95% CI: 1.68–5.13, *P* = .0002).

**Conclusion:**

The higher incidence of local tumor recurrence for surgical patients suggests that more prospective studies are needed to clarify outcomes for treatment of 1-4 metastasis less than 4 cm diameter.

**Key Points**

- Upfront resection vs radiation for a median number of brain metastases ≤ 4.
- First meta-analysis to focus on brain lesions with defined size and number.
- Surgical resection associated with worse local tumor recurrence.

**Importance of the Study**

It is important to obtain consensus on the best treatment for 1–4 brain metastases, which are often the first sign of CNS invasion by systemic cancers. Exclusive radiotherapy is preferred against a lesion < 2 cm in diameter, while surgical resection is often employed prior to radiation against larger tumors > 3 cm. However, the indications for mid-sized tumors roughly 1–3 cm in maximal diameter are not defined. Previous meta-analyses comparing surgical resection versus exclusive radiotherapy for treatment of brain cancer have not specified a number or size of metastases. By focusing on studies with a median number of brain metastases ≤ 4 of median diameter ≤ 4 cm, we observe that radiotherapy alone can exhibit better local control than when surgical resection was used prior to radiotherapy. This result summons the need for more prospective investigations into surgical treatment of a limited number of intermediately sized brain metastases.

Brain metastases arise in 10–30% of all cancer patients, with the majority originating from lung, breast, colorectal, renal cell carcinomas, and melanoma.^1,2^ Radiotherapy with or without preceding surgical resection (SR) is the standard treatment of care.^3^ However, there is a lack of consensus on whether to employ SR in the event of a solitary brain metastasis.^4^ The few available prospective studies that compare surgical and radiotherapy treatments of a single brain metastasis are low-powered and offer conflicting conclusions as to whether SR provides a survival benefit.^5–9^ Furthermore, current practice demonstrates opposing trends in the management of a specifically small, single metastasis: while smaller metastases (<2 cm diameter) are increasingly treated with stereotactic radiosurgery (SRS), the lone existence of a single metastasis often prioritizes it for SR.^3,4^ Accordingly, we used meta-analysis to compare the outcomes of SR followed by radiotherapy versus radiotherapy alone in the management of patients with a median number of brain metastases ≤4 of median diameter ≤4 cm. We chose these parameters for number and size based on the available literature that addresses our question.

Radiotherapy for brain metastases can be most suitable to target many, smaller lesions with diameter <4 cm.^10,11^ Whole-brain radiotherapy (WBRT) was the historically dominant radiation treatment modality until more targeted SRS techniques demonstrated less white matter toxicity and cognitive decline.^12–15^ Although WBRT has been recognized for its improved control of distant tumor recurrence compared to SRS alone,^16^ hypofractionated dosing schedules of SRS can further mitigate side-effects such as radiation necrosis and recurrence.^17^ Currently, WBRT is reserved for patients who are unable to undergo SR or SRS.^18^

Tumor size and multiplicity along with other considerations may complicate the choice between radiotherapy or surgical resection; in these instances, a multidisciplinary approach is often recommended. For large tumors in patients with good performance status and controlled primary and systemic disease, SR is most clearly indicated to immediately relieve a brain metastasis accompanied by mass effect, edema, and/or hydrocephalus.^18^ SR may also be chosen in cases where the lesion is suspected to be radioresistant, as can often occur with metastases from melanoma, renal cell carcinoma, and sarcoma.^19^ Following resection, irradiation of the brain after resection either with SRS or WBRT limits tumor recurrence and prolongs survival.^3,20^

Prevention of local recurrence is another key outcome in the management of metastases to the central nervous system. Although the mechanisms of recurrence have not been fully elucidated, extent of resection is highly correlated with recurrence and prognosis.^21^ While subtotal resection leaves a nidus of tumor cells available for future growth, the dispersion of microscopic tumor cells or their oncogenic contents is another potential mechanism by which the brain can be seeded for recurrent cancer.^22,23^ In support of this, piecemeal tumor resection has been associated with greater leptomeningeal dissemination compared to en bloc removal.^24,25^ Likewise, pachymeningeal seeding was found to be more common when metastases were managed with SR followed by SRS than when treated with radiation alone.^26^

Surgeons employ their expertise and judgment to weigh tumor size, multiplicity, and patient comorbidities when determining the treatment of metastatic brain cancer. Unfortunately, the indications for when to pursue surgery rather than radiosurgery alone are not clearly standardized. Accordingly, we undertook meta-analysis of reports that examined cases of a median number of brain metastases ≤4 of median diameter ≤4 cm. Indeed, the management of mid-sized tumors carries the least consensus as to whether surgical or exclusive radiotherapies are indicated.^4^

## Materials and Methods

### Literature Search

**Figure 1.**
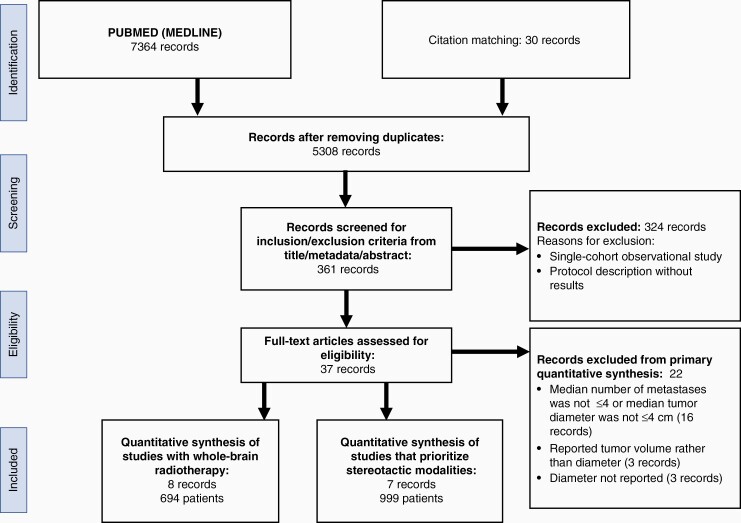
PRISMA flow diagram illustrating how records were identified and selected.

A systematic search following PRISMA (Preferred Reporting Items for Systematic Reviews and Meta-Analysis) guidelines was performed. A detailed protocol, including the inclusion, exclusion criteria, and search plan, were preregistered with PROSPERO (CRD42021242434). On February 18th, 2021, searches were performed on PUBMED, Web of Knowledge, and Cochrane Library for randomized controlled trials and observational studies written in the English language (Figure 1). The search strategy on PUBMED included: ((surgery) OR (surgical) OR (resection) OR (resected) OR (microsurgery) OR (microsurgical)) AND ((radiation) OR (radiotherapy) OR (stereotactic) OR (radiosurgery) OR (irradiation) OR (WBRT) OR (SRS) OR (Gamma Knife) OR (CyberKnife) OR (LINAC)) AND ((brain) OR (cerebral)) AND ((metastasis) OR (metastases) OR (metastatic)) NOT (case report) NOT (systematic review) NOT (meta-analysis) NOT (meta analysis) NOT (chemotherapy) NOT (immunotherapy). Studies were included in the primary analysis if they compared surgery to radiotherapy for a median number of brain metastases ≤4 with a median tumor diameter ≤4 cm. The number of 4 metastases with a 4 cm diameter threshold was selected to address our question with the maximum number of studies; we observed that many studies compared tumors either larger or smaller than 4 cm. Studies that included patients with a greater median number of brain metastases, and single-cohort observational studies were excluded. Studies that exclusively studied radioresistant metastases such as from melanoma^27,28^ were excluded. Ultimately, the studies we included feature a mix a of metastases arising from lung, breast, colon, melanoma, and kidney (Tables 1 and 2). Additionally, a few studies that only reported tumor volume rather than diameter were excluded since no reliable method to calculate a maximum tumor diameter from its volume could be found.^29–31^

**Table 1.** Patient and Treatment Characteristics of the Studies That Were Used in the Meta-Analysis of Studies That Prioritize Stereotactic Radiosurgery

First author, year	Treatment modality	Age (years)	Tumor diameter (cm)	Number of metastases	Primary cancer type (%)	Histology (%)	Radiation dose	Study length (months)	NOS
Churilla, 2019	Surgery (*n* = 59)	Median: 61.2	Range: 0.4–4	1-2	Breast (8.8), Colorectum (13.2), Kidney (7.9), Lung (58.8), Melanoma (2.6), Other (8.8)	Adenocarcinoma (60.5), Squamous (13.2), Melanoma (2.6), Anaplastic (0.9), Nonsmall cell (15.8), Other (7.0)	NA	64	See Cochrane Risk of Bias (Supplementary Figure S1)
	SRS (*n* = 75)	Median: 60.6	Range: 1–4		Breast (11.7), Colorectum (6.5), Kidney (11.0), Lung (59.1), Melanoma (5.8), Other (5.8)	Adenocarcinoma (53.2), Squamous (14.3), Melanoma (5.8), Anaplastic (1.9), Nonsmall cell (18.2), Other (6.5)	NA		
Lamba, 2019	surgery + SRS/SRT (*n* = 19)	56 ± 12	Median: 1.8 Interquartile: 1.4–1.9	1	Lung (21), Breast (16), Melanoma (32), Other (32)	NA	18–20 Gy	120	8
	SRS/SRT (*n* = 67)	63 ± 12	Median: 1.0 Interquartile: 0.6–1.3		Lung (43), Breast (13), Melanoma (19), Other (24)	NA	18–20 Gy		
Minniti, 2019	Surgery + Multifrac-SRS (*n* = 95)	Median: 59.4 Range: 26–80	Median: 3.3 Range: 1.6–4.8	1–4 (31% of cases had single met)	NA	Adenocarcinoma (78.9), Nonadenocarcinoma (21.1)	3 × 9 Gy	72	9
	Multifrac-SRS (*n* = 127)	Median: 61.1 Range: 34–83	Median: 3.0 Range: 1.7–4.3	1–4 (26% of cases had single met)	NA	Adenocarcinoma (85.8), Nonadenocarcinoma (14.2)	1 × 18–22 Gy		
Prabhu, 2017	Surgery + SRS (*n* = 48 matched, 157 total)	Median: 58 IQR: 48–66	Median gross total volume: 7.91 cm^3^ Interquartile: 6.2–15.4 cm^3^	1 met (68.2% of cases) 2 mets (22.3% of cases) 3 mets (8.3% of cases) >3 mets (1.2% of cases)	Lung (37.5), Breast (18.8), Melanoma (16.7), Renal (4.2), GI (6.3), Other (16.7)	NA	Median marginal dose: 15 Gy Interquartile: 15–18 Gy	18	9
	SRS (*n* = 48 matched; 66 total)	Median: 59.5 IQR: 51–68	Mean: 2.25 cm Median gross total volume: 6.27 cm^3^ Interquartile: 4.8–9.3 cm^3^	1 met (50% of cases) 2 mets (21.2% of cases) 3 mets (22.7% of cases) >3 mets (6% of cases)	Lung (37.5), Breast (31.3), Melanoma (8.3), Renal (8.3), GI (8.3), Other (6.3)	NA	Median marginal dose: 18 Gy Interquartile: 16.5–18 Gy		
Zimmerman, 2016	Surgery + Gamma Knife (*n =* 33)	Median: 62 Range: 43–80	Median: 4.0 Range: 3.0–6.8	Median: 1 Range: 1–6	Nonsmall cell lung cancer (42), Small-cell lung cancer (6), Breast (9), Radioresistant renal/melanoma/colon/sarcoma (33), Unknown/Other (9)	NA	Median: 15 Gy Range: 13–15 Gy	33	8
	Gamma Knife (*n* = 62)	Median: 66 Range: 29–92	Median: 3.5 Range: 3.0–5.8	Median: 2 Range: 1–16	Nonsmall cell lung cancer (32), Small-cell lung cancer (10), Breast (15), Radioresistant renal/melanoma/colon/sarcoma (29), Unknown/Other (19)	NA	Median: 15 Gy Range: 10–18 Gy	42	
Bougie, 2015	Surgery + radiation* (*n* = 43)	Median: 60 Range: 39–84	Median: 3.7 Range: 2.0–6.0	1	NA	Adenocarcinoma (74), Epidermoid (9), Large cell (5), Undifferentiated (12)	NA	70	7
	SRS (*n* = 72)	Median: 62 Range: 42–86	Median: 2.0 Range: 0.6–3.2			Adenocarcinoma (65), Epidermoid (13), Large cell (6), Undifferentiated (16)	Median max dose: 36 Gy (range: 20–48 Gy) Median marginal dose: 18 Gy (range: 12–24 Gy)	65	
Wagner, 2014	Surgery + SRS (*n* = 31)	Mean: 61.3 Range: 21.4–89.0	Median: 1.7	1	Breast (9), Nonsmall cell lung cancer (26), Renal cell carcinoma (21), Melanoma (61), Other (21)	NA	Median: 20 Gy	73	7
	SRS (*n* = 93)					NA		44	

* indicates varied modes of postsurgical radiation where 49% of cases had SRS, 23% had WBRT, 16% had SRS + WBRT, and 9% had no radiation. Newcastle-Ottawa Scale (NOS) was used to evaluate the quality of retrospective observational studies. NOS scores ≥ 6 are considered to be high quality. Abbreviations: NA, not available; SD, standard deviation; IQR, interquartile range; SRS, stereotactic radiosurgery; SRT, stereotactic radiotherapy.

**Table 2.** Patient and Treatment Characteristics of the Studies That Were Used in the Meta-Analysis of Studies That Utilize Whole-Brain Radiotherapy

First author, year	Treatment modality	Age (years)	Tumor diameter (cm)	Primary Cancer (%)	Histology (%)	Radiation dose	Study length (months)	NOS
Rades, 2011	Surgery + WBI (*n* = 111)	≤ 60:*n* = 56 > 60:*n* = 55	≤ 2.5 cm max diam: n = 53 > 2.5 cm max diam: n = 58	Breast cancer (22), Nonsmall cell lung cancer (49), Other (30)	Nonsmall cell lung cancer (49)	5 × 4 Gy: *n* = 13 10 × 3 Gy: *n* = 61 20 × 2 Gy: *n* = 37	NA	8
	WBI + RS (*n* = 41)	≤ 60:*n* = 22 > 60: *n* = 19	≤ 2.5 cm max diam: n = 22 > 2.5 cm max diam: n = 19	Breast cancer (24), Nonsmall cell lung cancer (44), Other (32)	Nonsmall cell lung cancer (44)	5 × 4 Gy: *n* = 5 10 × 3 Gy: *n* = 22 20 × 2 Gy: *n* = 14 median RS dose: 21 Gy		
Roos, 2011	Surgery + WBRT (*n* = 10)	Median: 58 Range: 43–72	Median: 2.4 Range: 1–4	Lung (50), Colorectal (20), Other (30)	NA	30 Gy in 10 fractions	54	See Cochrane Risk of Bias (Supplementary Figure S1)
	RS + WBRT (*n* = 11)	Median: 63 Range: 44–84	Median: 1.7 Range: 0.7–3.6	Lung (45), Colorectal (18), Other (36)	NA	Diameter ≤ 2 cm: 20 Gy Diameter = 2.1–3 cm: 18 Gy Diameter = 3.1–4 cm: 15 Gy	64	
Jalvakar, 2010	Surgery + WBRT (*n* = 24)	Median: 58	Mean: 2.8 Median: 3.0 Range: 1.5–3.5	Lung (54), Breast (17), Colon (11), Esophagus (9), Kidney (3.5), Melanoma (3.3), Bladder (3.3)	Melanoma (3.3)	WBRT: 30 Gy per 10 fractions	47	8
	Gamma knife (*n* = 11)	Median: 60	Mean: 2.0 Median: 2.2 Range: 0.74–3.3			Median: 33.5 Gy	43	
Muacevic, 2008	Surgery + WBRT (*n* = 33)	Mean ± SD: 58.3 ± 13.1 Median: 59 Range: 32–75	Mean ± SD: 2.4 ± 0.6 Median: 2	Lung (36.4), GUT (12.1), GIT (9), Melanoma (15.2), Breast (15.2), Liver (3), Unknown (9)	Melanoma (15.2)	2 Gy × 20 fractions	45	See Cochrane Risk of Bias (Supplementary Figure S1)
	Gamma knife (*n* = 31)	Mean ± SD: 58.3 ± 13.1 Median: 59 Range: 32–75	Mean ± SD: 2.1 ± 0.8 Median: 2	Lung (32.3), GUT (19.4), GIT (3.2), Melanoma (12.9), Breast (19.4), Liver (3.2), Unknown (9.7)	Melanoma (12.9)	Mean: 21 Gy Range: 14–27 Gy		
O’Neil, 2003	Surgery + WBRT (*n* = 74)	Mean ± SD: 62 ± 12 Median: 63 IQR: 55–70	≤ 3.5	Lung (54), GU (14), GI (9), Melanoma (8), Other (15)	Melanoma (8)	NA	98	8
	Radiosurgery + WBRT (*n* = 23)	Mean ± SD: 61 ± 13 Median: 66 IQR: 51–71		Lung (48), GU (17), GI (13), Melanoma (4), Other (17)	Melanoma (4)		108	
Schöggl, 2000	Surgery + WBRT (*n* = 66)	Median: 60	< 3 cm max diam. median: 12 500 mm^3^	Lung (24), Breast (14), Melanoma (15), Renal cell carcinoma (3), Colorectal carcinoma (17), Adenocarcinoma (20), Other (7)	Melanoma (15), Adenocarcinoma (20)	postop: 30 Gy/10 days	56	8
	Gamma-knife + WBRT (*n* = 67)	Median: 58	< 3 cm max diam. median: 7800 mm^3^	Lung (52), Breast (11), Melanoma (4), Renal cell carcinoma (11), Colorectal carcinoma (6), Adenocarcinoma (9), Other(7)	Melanoma (4), Adenocarcinoma (9), Other	median SRS: 17 Gy 30 Gy/10 days	44	
Muacevic, 1999	Surgery + WBRT (*n* = 52)	Mean ± SD: 56.3 ± 10.6 Median: 59 Range: 17-74	Mean ± SD: 2.7 ± 0.7 Median: 3	Lung (32.7), Genitourinary tract (19.2), Gastrointestinal tract (13.5), Melanoma (13.5), Breast (11.5), Other (1.9), Unknown (7.7)	Nonsmall cell bronchial carcinoma (32.7), Melanoma (13.5), Other (1.9), Unknown (7.7)	50 Gy/5 weeks	survival: 30.25 local recur: 23.8	9
	Gamma knife (*n* = 56)	Mean ± SD: 59.3 ± 13.7 Median: 59 Range: 27-83	Mean ± SD: 2.07 ± 0.9 Median: 2	Lung (30.4), Genitourinary tract (14.3), Gastrointestinal tract (5.4), Melanoma (28.6), Breast (14.3), Other (1.8), Unknown (5.4)	Nonsmall cell bronchial carcinoma (30.4), Melanoma (28.6), Other (1.8), Unknown (5.4)	Min dose mean: 21 Gy Max dose mean: 41 Gy	survival: 26.25 local recur: 23.8	
Mintz, 1996	Surgery + WBRT (*n* = 41)	Mean ± SD: 58.9 ± 8.98	Max ± SD: 2.54 ± 1.24	Lung (56), Breast (4.9), Colon or rectum (24.4), Skin (4.9), Renal (2.4), Other (2.4), Unknown (4.9)	Nonsmall cell (56)	3 × 10 Gy	18	See Cochrane Risk of Bias (Supplementary Figure S1)
	WBRT (*n* = 43)	Mean ± SD: 58 ± 9.86	Max ± SD: 2.96 ± 1.44	Lung (53.6), Breast (19.5), Colon or rectum (7.3), Skin (4.9), Renal (4.9), Head and neck (2.4), Other (7.3), Unknown (4.9)	Nonsmall cell (53.6)	3 × 10 Gy		

Newcastle-Ottawa Scale (NOS) was used to evaluate the quality of retrospective observational studies. NOS scores ≥ 6 are considered to be high quality. Abbreviations: NA, not available; SD, standard deviation; IQR, interquartile range; WBI, whole-brain irradiation; WBRT, whole-brain radiotherapy; RS, radiosurgery.

### Data Extraction

Prior to quantitative analysis, authors (DTK, AN, SSK, MJ) independently assessed articles for eligibility, and the corresponding author (WH) was consulted to resolve conflicts. Instances of occurrence were recorded for all available parameters such as all-cause mortality, and local recurrence at one-year and two-year time intervals. Local recurrence indicates intracranial tumor regrowth within the original tumor bed that had undergone surgical resection and/or radiotherapy. Local recurrence is distinct from distant recurrence, which refers to intracranial tumor regrowth outside of the original tumor bed. When two-year data was not available, parameters were calculated at the termination of each study. In the cases that studies did not explicitly report these rates, Kaplain-Meier curves were utilized to extract this data. WebPlotDigitizer v.4.4^32^ was used to import the image files of the curves, and the values at one- and two-year intervals were obtained for mortality and recurrence. Upon completion of data extraction, each parameter was independently validated by DTK and AN for accuracy.

### Statistical Analysis

A frequentist meta-analysis was performed using the *meta* package in R. For each study, the effect size and standard error were calculated. Odds ratios were subsequently pooled using the DerSimonian-Laird method. A random effects model was utilized in the cases where heterogeneity was significant. Heterogeneity was determined using the Cochran Q value and the I^2^ value such that I^2^ > 50% was considered significantly heterogeneous.

### Assessment of Quality

Quality assessment for each study was performed by utilizing standard methods. For randomized controlled trials (RCTs), the Cochrane Risk of Bias tool was used (Supplementary Figure S1). For each of the domains of bias, the degree of risk was classified as “low,” “high,” or “unclear.” To assess the quality of nonrandomized case-control studies, the Newcastle-Ottawa Scale (NOS) was calculated. This standardized risk of bias assessment scores studies according to patient selection, comparability, and data gathering to provide a combined score from 0–9. Scores ≥ 6 are generally attributed to higher quality studies. The NOS assessment was performed independently by authors (DTK and AN) and are provided in Tables 1 and 2. With neither the Cochrane Risk of Bias nor with NOS did we identify any consistent forms of bias across the included studies.

## Results

From our initial pool of 361 records, 15 records were included for meta-analysis (Figure 1, Tables 1 and 2). Of these records, four studies were RCTs,^7–9,33^ while 11 were observational studies.^34–44^ The 15 studies provided a total of 1693 patients, with 848 patients undergoing surgical treatment and 845 receiving exclusive radiotherapy. While all studies compared surgical intervention followed by radiation to radiation alone, the methodologies of radiation varied. Five of these studies compared surgery followed by SRS to SRS. One study compared surgery versus SRS. One study compared surgery followed by varied modes of postsurgical radiation (49% of cases had SRS, 23% had WBRT, 16% had SRS + WBRT, 9% had no radiation) versus SRS alone. Three studies compared surgery followed by WBRT versus gamma knife radiosurgery. Three studies compared surgery followed by WBRT versus SRS followed by WBRT. One study compared surgery followed by WBRT versus WBRT alone. One study compared surgery followed by WBRT versus gamma knife radiosurgery followed by WBRT. One study compared surgery followed by SRS versus SRS alone. The studies were grouped according whether SRS or WBRT was the predominant radiation modality and are summarized in Tables 1 and 2, respectively. The Risk of Bias for the RCTs is shown in Supplementary Figure S1.

### Survival in Studies That Prioritize Stereotactic Radiation Modalities

**Figure 2.**
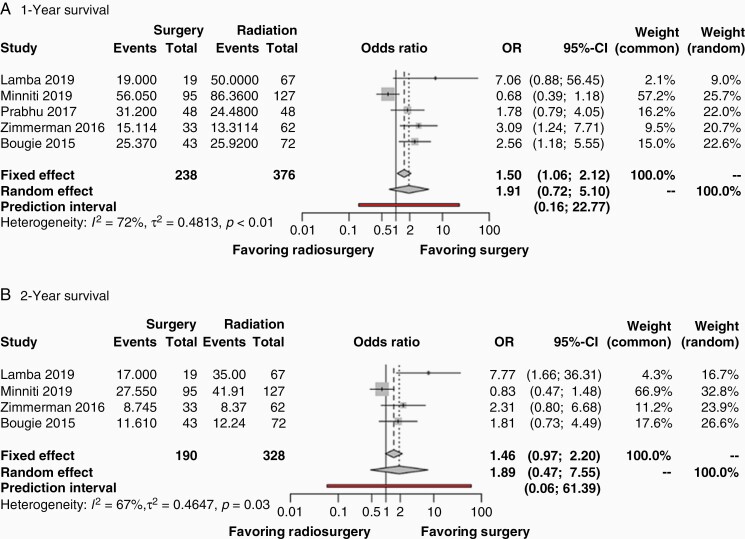
Forest plots of odds ratios from studies comparing the survival outcomes of patients undergoing initial surgical resection followed by SRS versus SRS alone in the treatment of a brain metastases (A.) one year after treatment, and (B.) two years after treatment. The contributing studies are summarized in Table 1. Abbreviations: OR, odds ratio; CI, confidence interval.

Figure 2 shows the results of a frequentist meta-analysis for survival outcome measurements of the studies in Table 1, which includes one RCT and six observational studies. One-year survival was reported by five of the studies with 238 patients in the surgical group and 376 patients in the nonsurgical group (Figure 2A). Significant heterogeneity was observed (I^2^ = 72%, *P* < .01), and a random effects model was used to perform the meta-analysis. This comparison between surgery + SRS versus SRS alone revealed a pooled OR of 1.91 (95% CI: 0.72–5.10, *P* = .14). The prediction interval was found to be 0.16–22.77. Two-year survival was reported by four studies, including 190 patients in the surgical group and 328 patients in the nonsurgical group (Figure 2B). Significant heterogeneity was observed (I^2^ = 67%, *P* = .03), and a random effects model was used to perform the meta-analysis. This comparison between surgery + SRS versus SRS alone revealed a pooled OR of 1.89 (95% CI: 0.47–7.55, *P* = .23). The prediction interval was found to be 0.06–61.39. Publication bias for survival measurements was nonsignificant for the one-year data but was significant for the two-year data as demonstrated by funnel plots in Supplementary Figure S2.

### Survival in Studies That Utilize Whole-Brain Radiotherapy

Supplementary Figure S3 show the results of a frequentist meta-analysis for survival outcomes in studies with WBRT from Table 2, which includes three RCTs and five observational studies. One-year survival (Supplementary Figure S3A) was reported by eight studies, including 411 total patients in the surgery cohort, and 283 patients in the nonsurgical cohort. Because of the presence of nonsignificant heterogeneity (I^2^ = 0%, *P* = .46), a fixed-effects model was used to perform the meta-analysis. This comparison between surgery + WBRT versus radiotherapy alone (either WBRT and/or SRS) revealed a pooled OR of 0.87 (95% CI: 0.63–1.20, *P* = .40). The prediction interval was found to be 0.59–1.31. Two-year survival (Supplementary Figure S3B) was reported by six studies, including 259 total patients in the surgery cohort, and 199 patients in the nonsurgical cohort. Nonsignificant heterogeneity was observed (I^2^ = 38%, *P* = .15), and a fixed-effects model was used to perform the meta-analysis. This comparison between surgery + WBRT versus radiotherapy alone (either WBRT and/or SRS) revealed a pooled OR of 1.18 (95% CI: 0.76–1.84, *P* = .46). The prediction interval was found to be 0.20–5.95.

### Local Recurrence in Studies That Prioritize Stereotactic Radiation Modalities

**Figure 3.**
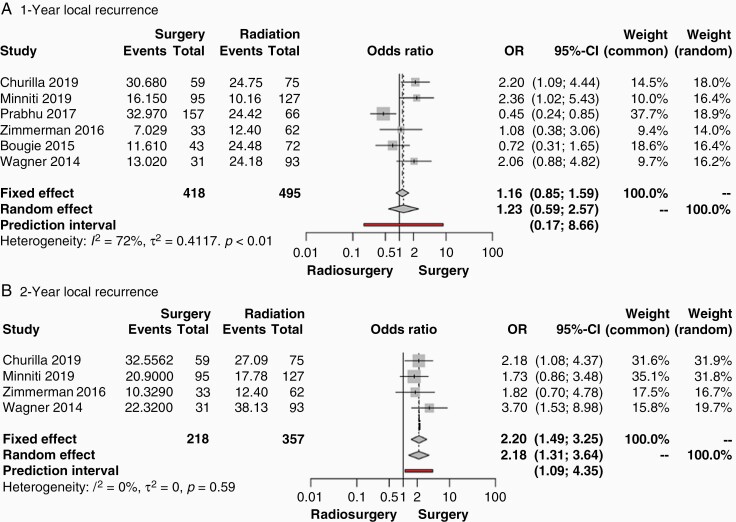
Forest plots of odds ratios from studies comparing the local tumor recurrence outcomes of patients undergoing initial surgical resection followed by SRS versus SRS alone in the treatment of brain metastases (A.) one year after treatment and, (B.) two years after treatment. The contributing studies are summarized in Table 1. Abbreviations: OR, odds ratio; CI, confidence interval; SRS, stereotactic radiosurgery.

Figure 3 shows the results of a frequentist meta-analysis for local recurrence measurements of the studies in Table 1. One-year local recurrence was reported by six of the studies with 418 patients in the surgical group and 495 patients in the nonsurgical group (Figure 3A). Significant heterogeneity was observed (I^2^ = 72%, *P* < .01), and a random effects model was used to perform the meta-analysis. This comparison between surgery ± SRS versus SRS alone revealed a pooled OR of 1.23 (95% CI: 0.59–2.57, *P* = .50). The prediction interval was found to be 0.17–8.66. Two-year local recurrence was reported by four studies, including 218 patients in the surgical group and 357 patients in the nonsurgical group (Figure 3B). Nonsignificant heterogeneity was observed (I^2^ = 0%, *P* = .59). Thus, a fixed effects model was used to perform the meta-analysis. This comparison between surgery ± SRS versus SRS alone revealed a pooled OR of 2.20 (95% CI: 1.49–3.25, *P* < .0001), indicating greater local recurrence associated with surgery. The prediction interval was found to be 1.09–4.35. Publication bias for survival measurements was nonsignificant as demonstrated by funnel plots in Supplementary Figure S2.

### Local Control in Studies That Utilize Whole-Brain Radiotherapy

**Figure 4.**
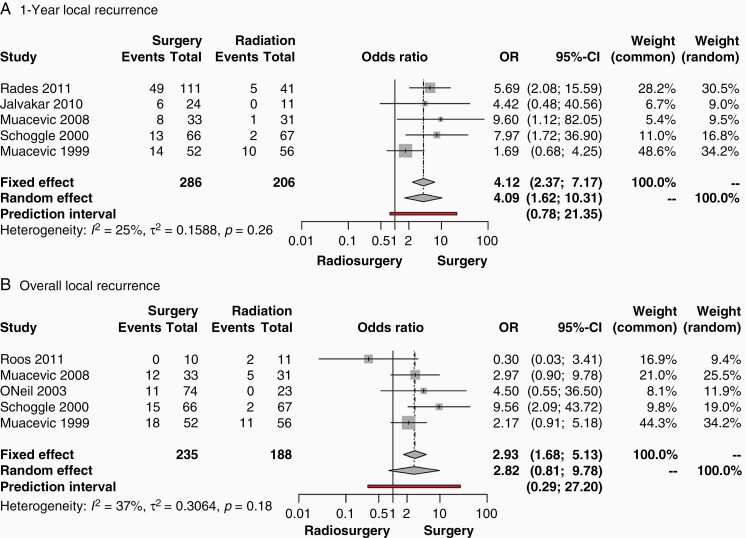
Forest plots of odds ratios from studies comparing the local tumor recurrence outcomes of patients undergoing initial surgical resection followed by radiation versus radiation alone in the treatment of brain metastases. (A.) one year after treatment and, (B.) at the end of study timepoint (Overall). The contributing studies are summarized in Table 2 and predominantly utilize whole-brain radiotherapy. Abbreviations: OR, odds ratio; CI, confidence interval; SRS, stereotactic radiosurgery.

Poor local control was defined as recurrence of tumor at the site of treatment. Local control was measured one year after treatment and at the termination of the studies since data two years after the procedure was not always reported. One-year local recurrence (Figure 4A) was reported by five studies, including 286 patients in the surgical cohort, and 206 patients in the nonsurgical cohort. Nonsignificant heterogeneity was observed (I^2^ = 25%, *P* = .26). Thus, a fixed-effects model was utilized to perform a meta-analysis. This comparison between surgery + WBRT versus radiotherapy alone (either WBRT and/or SRS) demonstrated a pooled OR of 4.12 (95% CI: 2.37–7.17, *P* < .0001), indicating greater local recurrence associated with surgery. The prediction interval was found to be 0.78–21.35. Five studies reported overall local recurrence at the concluding timepoint (Figure 4B), with a total of 235 patients in the surgical cohort, and 188 patients in the nonsurgical cohort. Because nonsignificant heterogeneity was observed (I^2^ = 37%, *P* = .18), a fixed-effects model was used to perform a meta-analysis. This comparison between surgery + WBRT versus radiotherapy alone (either WBRT and/or SRS) demonstrated a pooled OR of 2.93 (95% CI: 1.68–5.13, *P* = .0002), indicating greater local recurrence associated with surgery. The prediction interval was 0.29–27.20. Publication bias for local control measurements was nonsignificant as demonstrated by funnel and Labbe plots in Supplementary Figure S4.

## Discussion

The purpose of this meta-analysis was to compare surgical resection (SR) with postoperative radiotherapy versus radiotherapy alone in the initial treatment of a limited number of brain metastasis less than 4 cm in diameter. The presentation of a single brain metastasis less than 4 cm in diameter can present a conundrum, as current clinical intuition favors resection for a single lesion, but also favors exclusive stereotactic radiosurgery (SRS) for smaller lesions (<3-4 cm diameter) that do not exhibit mass effect against adjacent brain architectures.^3,4^ Furthermore, as systemic immunotherapies continue to prolong the survival of patients with brain metastasis, the outcomes of the initial surgical or radiotherapy step can reverberate across many years.

Our search yielded 15 studies that directly compare surgical against exclusive radiation therapy. Eight of these studies primarily relied upon whole-brain radiotherapy (WBRT) and were published between 1996–2011, while seven of the studies used SRS and were published between 2014–2019. Based on the key technological differences between WBRT versus SRS and the timeframes in which these modalities populate the literature search, we quantitatively analyzed the two sets of studies separately. While SRS is currently becoming the dominant form of radiotherapy even in cases with > 25 brain metastases,^45^ the studies with WBRT still met the inclusion criteria and provide an interesting comparison to the current standard of care.

Results from the meta-analysis of SRS cases included one RCT and six observational studies, and indicated no difference in one-year or two-year survival between patients who underwent SR followed by radiation versus patients who received radiotherapy alone (Figure 2). However, results showed greater local tumor recurrence in the surgical group compared to the exclusive radiotherapy group at the two-year but not at the one-year timepoint (Figure 3). This effect was not detected in a recent meta-analysis of studies with one or more metastases of unrestricted diameter.^46^ Indeed, by excluding any study that did not meet the criteria of a median number of brain metastases ≤4 with a median diameter ≤4 cm, we extracted an incidence of recurrence that may have been previously lost in the heterogeneity of disparate tumor sizes and number of metastases.

Since the greater local recurrence in the surgical arm was only observed after two years with only four contributing studies, it is possible that we observe an artifact of a subset of studies that recorded longer follow-up. This cannot be excluded, and ultimately points to the need for longer observation of patients in studies that address this question. Nevertheless, the surgical arm of WBRT studies also demonstrates greater local tumor recurrence both one year after surgery and at the terminal study timepoint (Figure 4). We intentionally separated SRS- and WBRT-based studies due to the global cerebral radiation exposure undergone in WBRT that can lead to dramatic cognitive decline. However, SRS and WBRT have been seen to offer comparable local control.^47^ Therefore, the higher surgical incidence of local recurrence in the WBRT studies aligns with and bolsters the same finding that we see in the surgical arm of SRS studies.

This meta-analysis underscores the importance of patient selection when considering surgical and radiation treatments in patients with few brain metastases of intermediate size. There are many instances when prompt surgical resection is advised.^48,49^ Tumors that demonstrate rapid growth, impose a mass effect on surrounding tissues, and/or precipitate edema are among the most urgent surgical candidates.^17^ Furthermore, a larger tumor (diameter > 4 cm) in a noneloquent location is typically removed by surgery before radiotherapy.^50^ Our present meta-analysis focused on the clinically ambiguous circumstance of 1-4 brain metastasis less than 4 cm in diameter. Findings suggest that although the surgical or pure radiation strategies offer no difference in survival, risk of local recurrence and tumor characteristics should be evaluated when considering a surgical strategy. Specifications of surgical technique, such as en bloc rather than piecemeal resection may abrogate this risk, but future trials are necessary to explore this question and to elucidate the mechanisms of micrometastasis.^24^

Extant studies vary in the reporting of tumor size and number, and subsequently draw mixed and inconclusive results. By establishing strict inclusion criteria of a median number of brain metastases ≤ 4 with a median tumor diameter ≤ 4 cm, our meta-analysis extracts a trend in local tumor recurrence that warrants further investigation. However, the trade-off of our strict inclusion criteria is a relatively small pool of 1693 patients from 15 total studies (including four RCTs). With these numbers, our analysis is inherently underpowered. Although our local control statistics render *p*-values well below 0.05, some of our prediction intervals cross the threshold of a 1.0 odds ratio. Prediction intervals attempt to capture how treatment effects might vary across different settings, and do not detract from the significance of the *p*-value.^51^ As other reports have noted, it is difficult to recruit patients and surgeons for RCTs that randomize the initial intervention against brain metastasis to be surgery or radiation.

The findings of this meta-analysis also should be weighed against additional limitations. Overall, only four of the included studies were RCTs, while the other 11 were retrospective observational studies. Each of the 11 had a high cumulative NOS score, suggesting good design, but we cannot avoid the intrinsic bias that comes from the pooling of retrospective studies. Furthermore, selection bias may have amplified the incidence of local tumor recurrence in the surgical arm: since rapid tumor growth and mass effect often prompt surgical intervention, recurrence may reflect the aggressive nature of these tumors rather than the treatment modality.

This meta-analysis provides an important contribution by pooling these studies, but well-designed randomized trials should be performed to adequately validate the findings. We also did our best to reduce selection bias and cross-validate the final paper selection, but may have missed some studies in our approach. Additionally, we had to exclude at least three studies that matched our criteria but reported tumor volume rather than diameter.^29–31^ Because not all tumors have a consistent shape, we elected not to estimate tumor diameter from the tumor volume.

Overall, this meta-analysis highlights the importance of future RCTs that compare the recurrence outcomes when treating 1–4 brain metastases initially with surgery versus radiation. When comparing the outcomes of survival and recurrence for brain metastases initially treated with SR or SRS, it is important to try to control for the patient population, the size, and location of the lesion, and the number of lesions since the presentation of two or more metastatic lesions in the brain carries a worse prognosis than a single lesion.^52^ Our meta-analysis showed no difference in survival between the two treatment strategies and indicates that radiotherapy alone was not inferior to SR in local tumor control.

Important questions remain regarding local tumor recurrence following treatment of brain metastases. Clinically, the predictive factors that predispose a patient to recurrence are a piecemeal resection and a tumor volume >9.7 cm^3^ of the single metastasis,^53,54^ along with subtotal resection.^55^ The microenvironment of a metastatic lesion likely influences recurrence following surgery and/or radiotherapy as these interventions disperse neoplastic contents. Tumor vascularization and epidermal growth factor receptor status, for example, may be important variables, as well as the molecular characteristics of the metastasis that may even differ from the primary cancer.^56,57^

## Conclusion

By focusing on a limited number of brain metastases ≤ 4 cm in diameter, our meta-analysis showed better local tumor control in patients treated with exclusive radiotherapy when compared to patients treated by surgical resection followed by radiotherapy. Patient selection is of paramount importance to identify surgical candidates.

## Supplementary Material

vdac033_suppl_Supplementary_Figures
