# Supplementary material for: Management of brain metastasis. Surgical resection versus stereotactic radiotherapy: a meta-analysis
Source: Neurooncol Adv. 2022 Mar 9;4(1):vdac033. doi: 10.1093/noajnl/vdac033 (PMC8982204; doi:10.1093/noajnl/vdac033)

Supplementary Figure S1. The Cochrane Collaboration Risk of Bias analysis of four randomized control trials. Green = low risk, Yellow = unclear risk, Red = high risk.

Year	Lead author	Random sequence generation (selection bias)	Allocation concealment (selection bias)	Blinding of participants and personnel (performance bias)	Blinding of outcome assessment (detection bias)	Incomplete outcome data (attrition bias)	Selective reporting (reporting bias)	Other bias
1996	Mintz	unclear risk	unclear risk	unclear risk	unclear risk	low risk	unclear risk	low risk
2008	Muacevic	low risk	low risk	unclear risk	unclear risk	high risk	unclear risk	low risk
2011	Roos	low risk	low risk	unclear risk	unclear risk	low risk	unclear risk	low risk
2018	Churilla	low risk	high risk	low risk	high risk	low risk	unclear risk	low risk

Supplementary Figure S2. Funnel Plots for Survival and Local Recurrence in the studies from Table 1 analyzing publication bias of data.

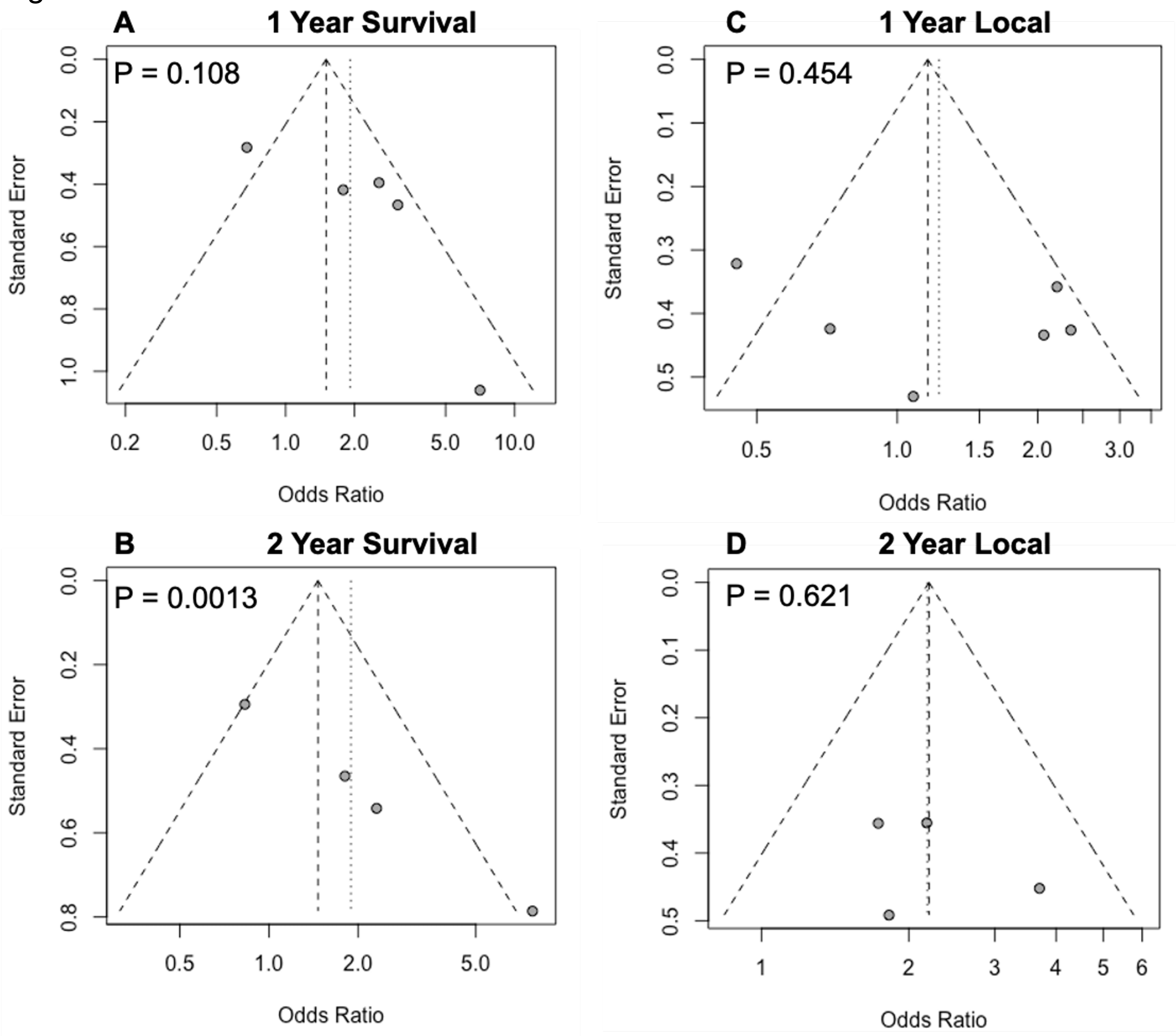

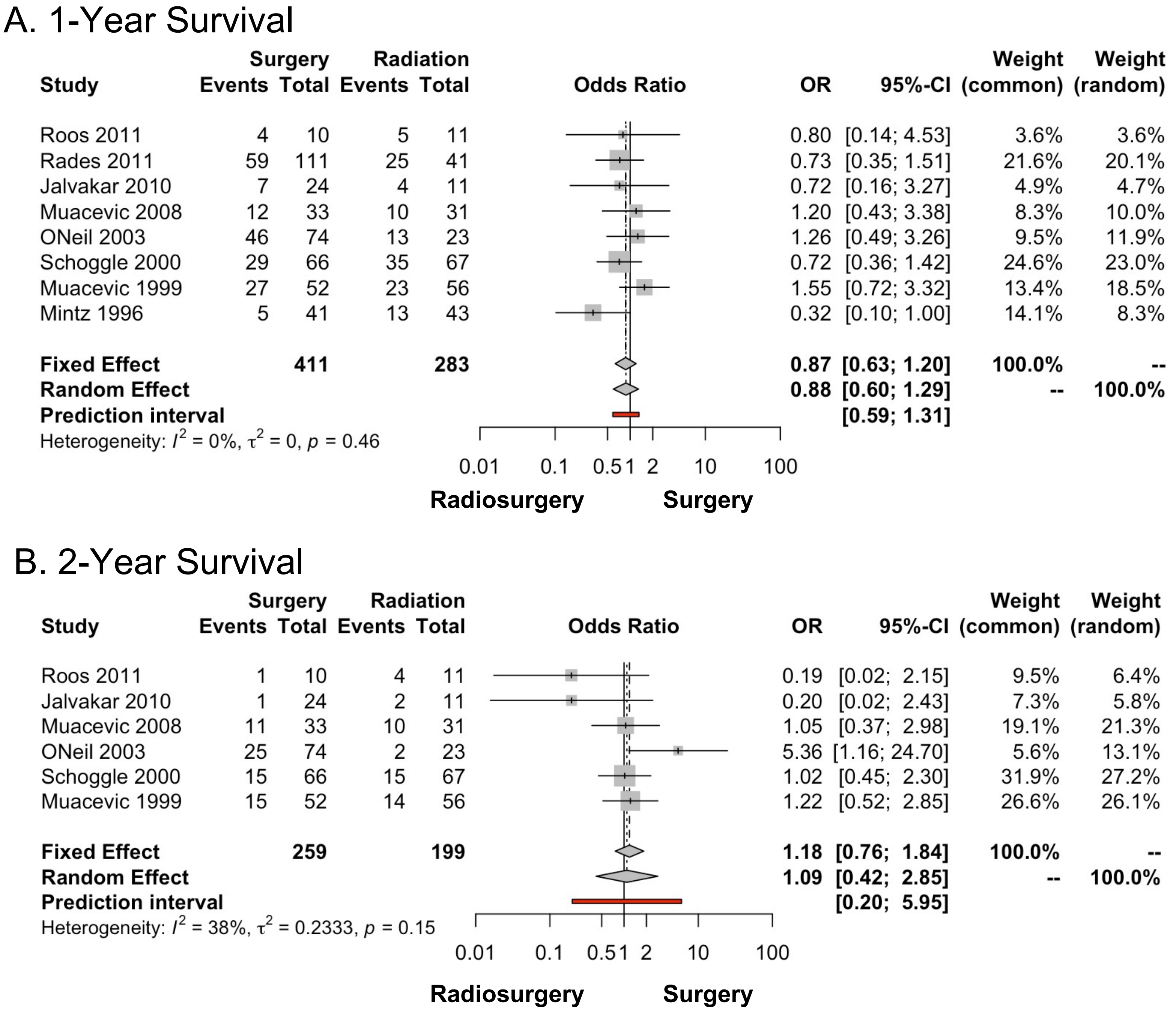

Supplementary Figure S3. Forest plots of odds ratios from studies comparing the survival outcomes of patients undergoing initial surgical resection followed by radiation versus radiation alone. The contributing studies are summarized in Table 2. Abbreviations: OR = odds ratio. CI = confidence interval.

Supplementary Figure S4.
Funnel and Labbe Plots for Local Recurrence in studies from Table 1 analyzing publication bias of data.

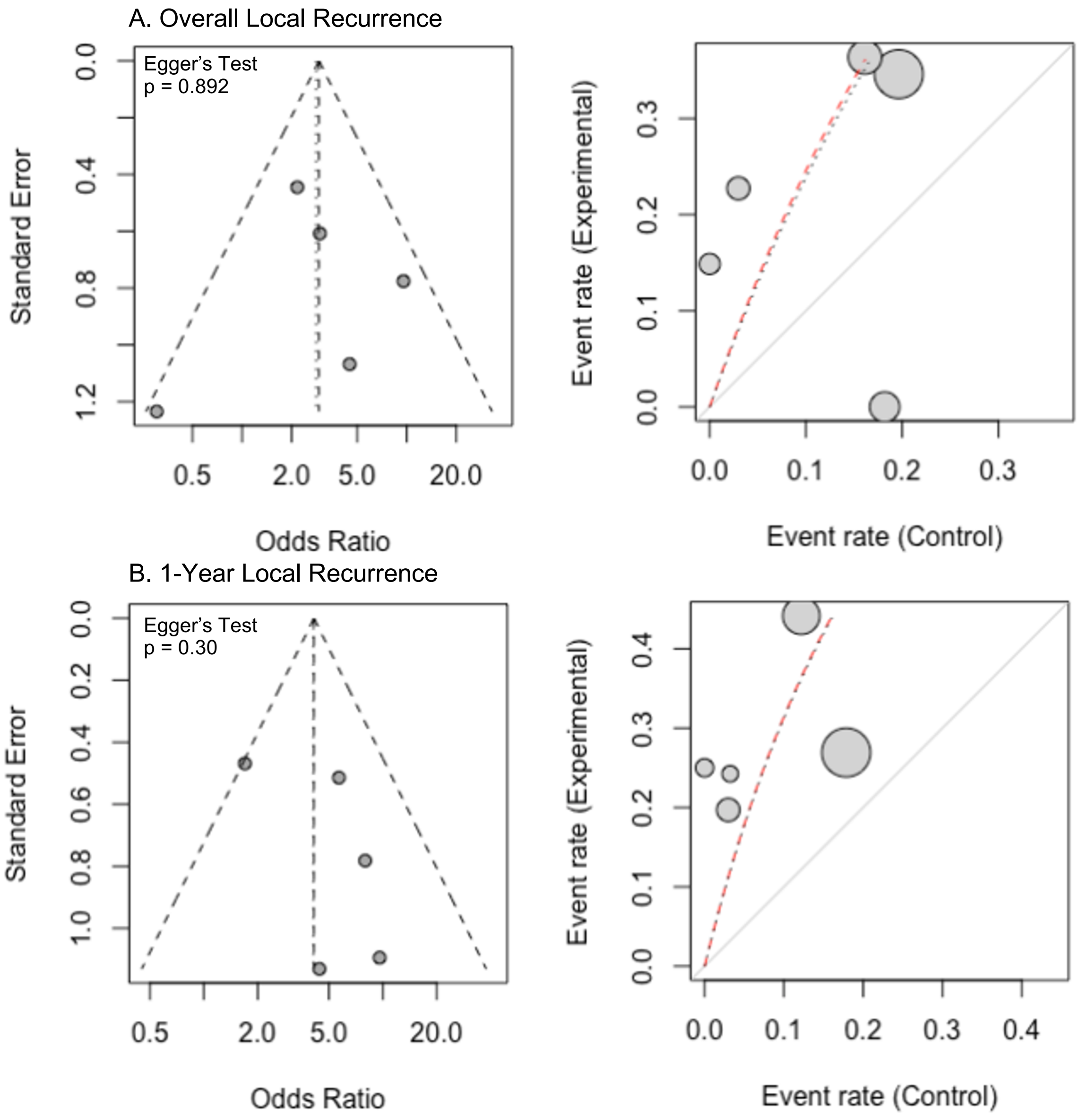

Supplement: vdac033_suppl_Supplementary_Figures [file vdac033_suppl_supplementary_figures.pdf]
